# Diabetes, hypertension and dyslipidemia medication prescribing in Qatari primary care settings: a retrospective analysis of electronic medical records

**DOI:** 10.1186/s40545-021-00353-4

**Published:** 2021-08-11

**Authors:** Mohamed Ahmed Syed, Ahmed Sameer Al Nuaimi, Hamda Abdulla A/Qotba, Abduljaleel Abdullatif Zainel, Tamara Marji, Uzma Razaq

**Affiliations:** 1 Directorate of Clinical Affairs, Primary Health Care Corporation, P.O. Box 26555, Doha, Qatar; 2 Directorate of Clinical Operations, Primary Health Care Corporation, P.O. Box 26555, Doha, Qatar

**Keywords:** Primary health care, Diabetes, Hypertension, Dyslipidaemia, Prescriptions, Qatar

## Abstract

**Background:**

Globally, non-communicable diseases (NCDs) are recognised as a leading cause of morbidity and mortality. Medications and medicines optimisation play an important role in the management of modifiable physiological risk factors and NCDs. The importance of lifestyle interventions in prevention of modifiable risk factors is also well established. The aim of this paper was to describe the quantity of type 2 diabetes mellitus (T2DM), hypertension and dyslipidaemia prescribing in Qatari primary care settings. Its findings will provide necessary information to inform pharmaceutical policy and practice.

**Methods:**

The study was undertaken in Qatar’s publicly funded primary health care centres. Data sources for this study comprised electronic medical records. The Anatomical Therapeutic Chemical (ATC) drug classification system was used to classify the medications prescribed. The number and proportion of medications by age, sex, nationality and diagnosis (T2DM, hypertension and dyslipidaemia) were reported.

**Results:**

A total 81,569 individuals were included (18–29 years 2.4%; 30–39 years 11.7%; 40–49 years 25.4%; 50–59 years 31.9% and ≥ 60 years 28.6%). 55.6% participants were male. On average 10.2 medications were prescribed per person and 2.3 medications were included in each prescription. T2DM medications were most prescribed (*N* = 361,87780,799; 43.2%) followed by hypertension (*N* = 303,086; 36.2%) and dyslipidaemia (*N* = 172,163; 20.5%). Of the total medications prescribed, 72% (*N* = 605,488) were prescribed in individuals aged 50 years and above. Men were prescribed 62% (*N* = 515,043) medications while women were prescribed 38% (*N* = 322,083) medications. Southern Asians (*N* = 330,338; 39%) were prescribed most medication followed by Qataris (*N* = 181,328; 22%) and Northern African (*N* = 145,577; 17%).

**Conclusions:**

In Qatar’s primary care settings, average medications prescribed per patients were found to be higher compared to other populations. While medications were actively prescribed for the 3 conditions, the study found variations by medication type, age, gender and nationality. Rational guidelines for the utilisation of medications need to be established with the support of real-world evidence.

**Supplementary Information:**

The online version contains supplementary material available at 10.1186/s40545-021-00353-4.

## Background

Globally, non-communicable diseases (NCDs) are recognised as a leading cause of morbidity and mortality. The burden of NCDs continues to increase in most countries. Aging, demographic transition, lifestyle changes, globalisation and urbanisation are factors that contribute to the increased prevalence, severity and complexity of NCDs [1].

Medications play a crucial role in maintaining health, preventing illness, managing chronic conditions [2]. However, there is a growing body of evidence suggesting a need to improve medicines use [2]. It is estimated that only 4%–21% of patients in primary care receive optimum benefit from their medicines [3]. ‘Medicines optimisation’ can be described as a system of processes and behaviours that determine how medicines are used in a health system for the safe and effective use of medicines to enable the best possible outcomes [4]. The goal of medicines optimisation is to help patients to improve their outcomes, take their medicines correctly, avoid taking unnecessary medicines, reduce wastage of medicines and improve medicines safety [5].

In Qatar, as in other countries, NCDs are a concern. A recent study reported 16.2% of the population in publicly funded primary care settings were diagnosed with one or more NCDs [6].

Modifiable physiological risk factors for NCDs were also reported to be high—insulin resistance (60.3%), hypertension (53.5%) and dyslipidaemia (48.5%) [7]. Differences in prevalence by age, sex and nationality were reported [6, 7]. The importance of lifestyle interventions in prevention of modifiable risk factors is well established [8–10]. Medications play an important role in the management of modifiable physiological risk factors and NCDs. Therefore, it is essential to optimise medications and encourage lifestyle interventions to improve health outcomes tailored to the population. This study aims is to describe the volume of type 2 diabetes mellitus (T2DM), hypertension and dyslipidaemia medications by age, sex and nationality in Qatari primary care settings. The study’s findings provide necessary information to inform pharmaceutical policy and practice.

## Methods

### Patient and public involvement

Patients or the public were not involved in the design of the study.

### Study setting

Qatar is a sovereign and independent state in the Middle East with a modern publicly funded healthcare system for Qataris and expatriates. This includes a universal primary care delivered by the Primary Health Care Corporation (PHCC), the largest primary care provider in the country. As of June 2020, a total of 1,461,759 individuals (approximately 70% of the country’s population) were registered across 27 PHCC health centres. Of these, 421, 283 individuals aged ≥ 18 visited a health centre at least once between 1st January 2017 and 31st December 2017. All care is free of charge except medications which are heavily subsidised by the government.

### Study population and data collection

Data source for this study comprised PHCC electronic medical records (EMR). The required data were extracted from the PHCC’s EMR. PHCC has a single EMR system across all PHCC health centres. The study population  included individuals meeting all of the following criteria (1) aged ≥ 18 (2); visited a health centre between 1st January 2017 and 31st December 2017; (3) had a diagnosis of T2DM, hypertension and/or dyslipidaemia and (4) were prescribed one or more medication for a diagnosis of T2DM, hypertension or dyslipidaemia.

### Definitions and data analysis

For the purposes of this study, a ‘clinical encounter’ was defined as a clinical interaction between a patient and PHCC physician resulting in a prescription of a medication for one or more of the three diagnosis targeted in the study. Medications were defined as ‘any chemical classified as a pharmaceutical compound under the Anatomical Therapeutic Chemical (ATC) classification and prescribed (as unique or refill prescriptions) to a patient for any one of the three diagnosis in a clinical encounter’ while prescription were defined as ‘one or more medications prescribed for a specific diagnosis in a clinical encounter’. A clinical encounter may have had 1, 2 or 3 prescriptions.

Variables for age, sex, nationality, diagnosis and medication prescribed for diagnosis were extracted from PHCC’s EMR in an anonymised manner by the health information management department which oversees data recorded in the EMR. ‘Statistical Package for the Social Sciences’ statistical software package was used to analyse data collected. To analyse the population characteristics (age, sex and nationality; see online Table A in Additional file 1) and medications prescribed for three diagnoses—hypertension, T2DM and dyslipidaemia—basic descriptive statistics were used. The ATC drug classification system was used to classify the medications prescribed. In the ATC drug classification system, the medications are divided into different groups according to the organ or system on which they act and their therapeutic, pharmacological and chemical properties [11]. For the purposes of this study, ATC level 4 (code indicating the chemical/therapeutic/pharmacological subgroup and consisting of one letter) was counted as one medication.

To ensure the accuracy of the data, a two-stage validation process was undertaken. The codes used to extract data from the EMR were developed by a health information management specialist and reviewed by another specialist to ensure its accuracy in extracting the required dataset. A total of 201 unique medications (by generic name) were included in the dataset. They were categorised into the ATC classification independently by two consultants. Any discrepancies in selection were resolved through discussions at a consensus meeting.

### Ethical approval

The data for the study were collected in an anonymised manner. None of the subjects’ personal information was available to the research team. Overall, the study was conducted with integrity according to generally accepted ethical principles and was reviewed and approved under the exempt review category by the PHCC’s Research-Subcommittee (PHCC/RS/18/02/003).

## Results

### Population characteristics

The study included a total 81,569 individuals who were diagnosed with one or more of the following—T2DM, hypertension and dyslipidaemia (see Table 1). Majority by nationality were Southern Asian or Qatari (*N* = 49,786; 61%) and by age over 40 years (*N* = 70,107; 85.9%).

**Table 1:** Population characteristics

	***N***	**%** (total *N* = 81,569)
**Age (years)**		
18–29	1939	2.4
30–39	9523	11.7
40–49	20,757	25.4
50–59	26,031	31.9
≥ 60	23,319	28.6
**Sex**		
Female	36,180	44.4
Male	45,389	55.6
**Nationality**		
Qatar	22,003	27.0
Northern Africa	13,404	16.4
Sub-Saharan Africa	947	1.2
Northern America	486	0.6
South-eastern Asia	5638	6.9
Southern Asia	27,783	34.1
Western Asia (excluding Qatar)	10,704	13.1
All others	603	0.7

### Overall medications prescribed

A total of 837,126 medications were prescribed using 359,079 prescriptions for 81,569 individuals (see Table 2). T2DM medications were most prescribed (*N* = 361,877; 43.2%) followed by hypertension (*N* = 303,086; 36.2%) and dyslipidaemia (*N* = 172,163; 20.5%). The most commonly prescribed medications were HMG-CoA reductase inhibitors [C10AA]- (also known as statins) (*N* = 169,301; 20.2%), biguanides [A10BA]- (*N* = 115,690; 13.8%), sulfonamides [A10BB]- (urea derivatives) (*N* = 88,935; 10.6%) and angiotensin-converting enzyme inhibitors [C09AA]- (plain) (*N* = 69,409; 8.3%) accounting for 52.9% (*N* = 443,335) of all medications prescribed (see Table 3).

**Table 2:** Prescriptions by diagnosis

Diagnosis	Prescriptions *N* (%)
Hypertension	145,546 (40.5)
T2DM	136,569 (38.0)
Dyslipidaemia	76,964 (21.4)
Total	359,079

**Table 3:** Overall medications prescribed

Diagnosis	Medication	ATC code	N	%
Hypertension	Angiotensin-converting enzyme inhibitors, plain	[C09AA]-	69,409	8.3
	Dihydropyridine derivatives	[C08CA]-	55,356	6.6
	Angiotensin II antagonists, plain	[C09CA]-	40,709	4.9
	Beta blocking agents, plain, selective	[C07AB]-	31,336	3.7
	Angiotensin-converting enzyme inhibitors and diuretics	[C09BA]-	24,463	2.9
	Angiotensin II antagonists and diuretics	[C09DA]-	21,569	2.6
	Angiotensin-converting enzyme inhibitors and calcium channel blockers	[C09BB]-	15,887	1.9
	Sulfonamides, plain (low ceiling diuretics)	[C03BA]-	15,143	1.8
	Angiotensin II antagonists and calcium channel blockers	[C09DB]-	14,710	1.8
	Sulfonamides, plain (high ceiling diuretics)	[C03CA]-	3779	0.4
	Alpha and beta blocking agents	[C07AG]-	3178	0.4
	Beta blocking agents, selective, and thiazides	[C07BB]-	2308	0.3
	Thiazides, plain	[C03AA]-	2038	0.2
	Beta blocking agents, plain, non-selective	[C07AA]-	1654	0.2
	Clonidine and analogues	[C02AC	849	0.1
	Methyldopa	[C02AB]-	698	0.1
	Total		303,086	36.2
T2DM	Biguanides	[A10BA]-	115,690	13.8
	Sulfonamides, urea derivatives	[A10BB]-	88,935	10.6
	Combinations of oral blood glucose lowering drugs	[A10BD]-	62,340	7.4
	Insulins and analogues for injection, long-acting	[A10AE]-	28,457	3.4
	Dipeptidyl peptidase 4 (dpp-4) inhibitors	[A10BH]-	25,246	3.0
	Insulins and analogues for injection, intermediate-acting combined with fast-acting	[A10AD]-	13,686	1.6
	Insulins and analogues for injection, fast-acting	[A10AB]-	11,594	1.4
	Thiazolidinediones	[A10BG	11,405	1.4
	Insulins and analogues for injection, intermediate-acting	[A10AC]-	2688	0.3
	Other oral blood glucose lowering drugs	[A10BX]-	1095	0.1
	Glucagon-like peptide-1 (GLP-1) analogues	[A10BJ]-	433	0.1
	Alpha glucosidase inhibitors	[A10BF]-	308	0.0
	Total		361,877	43.2
Dyslipidaemia	HMG-CoA reductase inhibitors	[C10AA]-	169,301	20.2
	Other lipid modifying agents	[C10AX]-	2679	0.3
	Fibrates	[C10AB]-	183	0.0
	Total		172,163	20.5
Grand total			837,126	

On average 10.2 medications were prescribed per person during the study period (3.7 hypertension medications per person; 4.3 T2DM medications; 2.1 dyslipidaemia medications per person) and 2.3 medications were included in each prescription (0.8 hypertension medication per prescription; 1 T2DM medication per prescription; 0.5 hypertension medication per prescription).

### Medications prescribed by age

Of the total medications (*N* = 837,126) prescribed, 72% (*N* = 605,488) were prescribed in individuals aged 50 years and above. In 18–29 year olds, 72% (*N* = 4,664) medications prescribed were for T2DM. Majority of individuals aged 30 years and above were prescribed T2DM medications (39.9%–53.5%) followed by hypertension (31.3.4%–39.4.1%) and dyslipidaemia medications (16.3%–21.7%) (see Table 4). Biguanides (29.7%) followed by insulins and analogues for injection (long-acting) (12.4%) and insulins and analogues for injection (fast-acting) (11.8%) were most prescribed medications in 18–29 year olds. In 30–39 year olds, biguanides (20.2%) followed by statins (16%) and sulfonamides (urea derivatives) (10.8%) were most prescribed. Statins (20%–21.3%) followed by biguanides (11.8%–15.5%) and sulfonamides (urea derivatives) (9.8%–11.6%) were most commonly prescribed in individuals aged 40 and above (see online Table B in Additional file 1).

**Table 4:** Medications prescribed by age

Diagnosis	18–29		30–39		40–49		50–59		≥ 60	
	(*N*)	(%)	(*N*)	(%)	(*N*)	(%)	(*N*)	(%)	(*N*)	(%)
Hypertension	1291	20	16,505	31.3	59,403	34.5	103,941	35.1	121,946	39.4
T2DM	4664	72.1	27,687	52.5	77,989	45.2	128,017	43.2	123,520	39.9
Dyslipidaemia	515	8	8586	16.3	34,998	20.3	64,112	21.7	63,952	20.7
Total	6470		52,778		172,390		296,070		309,418	

### Medications prescribed by sex

Men were prescribed 62% (*N* = 515,043) medications while women were prescribed 38% (*N* = 322,083) medications (see Table 5). T2DM medications were most commonly prescribed for both men and women (*N* = 229,596; 44.6% and *N* = 132,281; 41.1%, respectively) followed by hypertension (*N* = 178,544; 34.7% and *N* = 124,542; 38.7%, respectively) and dyslipidaemia (*N* = 106,903; 20.8% and *N* = 65,260; 20.3%, respectively) medications (see online Table C in Additional file 1).

**Table 5:** Medications by sex

	Female		Male	
	*N*	%	*N*	%
Hypertension	124,542	38.7	178,544	34.7
T2DM	132,281	41.1	229,596	44.6
Dyslipidaemia	65,260	20.3	106,903	20.8
Total	322,083		515,043	

### Medications prescribed by nationality

Southern Asians (*N* = 330,338; 39%) were prescribed most medication followed by Qataris (*N* = 181,328; 22%) and Northern African (*N* = 145,577; 17%) (see Table 6). The most commonly prescribed medication amongst South-eastern Asians (*N* = 25,335; 59.2%), Northern Americans (*N* = 1,921; 39.8%) and Western Asians (*N* = 45,342; 38%) was for hypertension. The most commonly prescribed medication amongst Southern Asians (*N* = 153,621; 46.5%), Qataris (*N* = 81,933; 45.2%), Northern Africans (*N* = 63,357; 43.5%) and sub-Saharan Africans (*N* = 3,548; 42.8%) was for T2DM (see online Table D in Additional file 1).

**Table 6:** Medications by nationality categories

	Qatar		Northern Africa		Sub-Saharan Africa		Northern America		South-eastern Asia		Southern Asia		Western Asia (excluding Qatar)		All others	
	*N*	%	*N*	%	*N*	%	*N*	%	*N*	%	*N*	%	*N*	%	*N*	%
Hypertension	61,210	33.8	52,468	36	3378	40.8	1921	39.8	25,335	59.2	111,389	33.7	45,342	38	2043	42.8
T2DM	81,933	45.2	63,357	43.5	3548	42.8	1577	32.7	9607	22.4	153,621	46.5	46,921	39.4	1312	27.5
Dyslipidaemia	38,185	21.1	29,752	20.4	1358	16.4	1325	27.5	7888	18.4	65,328	19.8	26,914	22.6	1413	29.6
Total	181,328		145,577		8284		4823		42,830		330,338		119,177		4768	

*Refer to additional file for nationality classification*

## Discussion

### Summary

The study found a total of 837,126 medications were prescribed using 359,079 prescriptions for 81,569 individuals, an average of 10.2 medications per patient. This is higher in comparison to a Swedish study which reported an average of 2.3 medications per person in chronic disease patients aged 25–70 years [12] and a Saudi Arabian study which reported an average of 6.4 medications per person in chronic disease patients aged 65–70 years [13]. This study, to the authors’ knowledge, is the first such comprehensive study in the Middle East. It adds valuable insight on prescribing patterns in primary settings for T2DM, hypertension and dyslipidaemia which have an increasing burden in Qatar and also in the Middle East [14]. Its findings will help optimise prescribing and medicines management in patients with one or more of the diagnosis.

### Strengths and limitations

The study’s strength includes a large sample and reliable data extracted from an EMR system. This facilitates development of strategies related to medicines and provide a baseline for future research.

The limitations of the study include the following: a cross-sectional study design which provides a snapshot in time. Furthermore, the study only included patients who were 18 years and above and those who attended a PHCC health centre in 2017. Therefore, the findings of the study cannot be considered comprehensive and nationally representative.

### Comparison with existing literature

The study found T2DM medications were most prescribed (43.2%) followed by hypertension (36.2%) and dyslipidaemia (20.5%). In terms of individual medication, statins were most commonly prescribed medications (20.2%). However, a recent study reported a gap between guidelines and practice in the management of dyslipidaemia in the Middle East [15]. Ensuring that individuals diagnosed with dyslipidaemia are prescribed medications in accordance to issued guidelines is essential. [7]. Similarly, medications for hypertension and insulin resistance should also be in line with issued guidelines as prevalence of hypertension was reported to be higher than dyslipidaemia—53% and 60.3% [7]. A review by Al-Maatouq et al. found metformin, a biguanide, to be used less frequently to initiate antidiabetic therapy in the Middle East than in other countries [16].

Seventy-two percent of medications prescribed were in individuals aged 50 and above in this study. Men were prescribed more medications than women (62% and 38%, respectively). In a Flemish-Belgian study, 54.8% of individuals aged 50 and above were prescribed medications. Men and women were prescribed almost similar amounts of medications (56.8% and 52.9%, respectively) [17]. Figures from this study show more medications were prescribed in individuals aged 50 years and above. Furthermore, men are prescribed more medication than women. This may be due to the fact that men have higher prevalence of non-communicable diseases compared to women in Qatar [6].

Southern Asians, Qataris and Northern African were prescribed most medications (39%, 22% and 17%). These nationalities were the largest nationality groups in the study and the same nationality groups were found to have the highest prevalence of metabolic syndrome [7] and non-communicable diseases in the population [6]. This is a possible explanation for the prescribing pattern seen in these nationality groups.

## Conclusions

In Qatar’s primary care settings, average medications prescribed per patients were found to higher compared to other populations. While medications are actively prescribed for the 3 conditions, study found variations by medication type, age, gender and nationality. Rational guidelines for the utilisation of medications need to be established with the support of real-world evidence.

## Supplementary Information

**Additional file 1.** Additional tables.

## Data Availability

The datasets used and/or analysed during the current study available from the corresponding author on reasonable request.

## Abbreviations

NCDs: Non-communicable diseases
T2DM: Type 2 diabetes mellitus
ATC: Anatomical Therapeutic Chemical
EMR: Electronic medical records
PHCC: Primary Health Care Corporation
WHOCC: World Health Organization Collaborating Centre for Drug Statistics
