# Supplementary material for: Diabetes, hypertension and dyslipidemia medication prescribing in Qatari primary care settings: a retrospective analysis of electronic medical records
Source: J Pharm Policy Pract. 2021 Aug 11;14:67. doi: 10.1186/s40545-021-00353-4 (PMC8356399; doi:10.1186/s40545-021-00353-4)
Supplement: Supplementary file 1 — Additional file 1. Additional tables. [file 40545_2021_353_MOESM1_ESM.docx]

Table A: Nationality classification by region

| **Nationality Classification** | |
| --- | --- |
| **Region** | **Country** |
| Northern Africa | \| Algeria \| \| --- \| \| Egypt \| \| Libya \| \| Morocco \| \| Sudan \| \| Tunisia \| \| Western Sahara \| |
| Sub-Saharan Africa | \| British Indian Ocean Territory \| \| --- \| \| Burundi \| \| Comoros \| \| Djibouti \| \| Eritrea \| \| Ethiopia \| \| French Southern Territories \| \| Kenya \| \| Madagascar \| \| Malawi \| \| Mauritius \| \| Mayotte \| \| Mozambique \| \| Réunion \| \| Rwanda \| \| Seychelles \| \| Somalia \| \| South Sudan \| \| Uganda \| \| United Republic of Tanzania \| \| Zambia \| \| Zimbabwe \| \| Angola \| \| Cameroon \| \| Central African Republic \| \| Chad \| \| Congo \| \| Democratic Republic of the Congo \| \| Equatorial Guinea \| \| Gabon \| \| Sao Tome and Principe \| \| Botswana \| \| Eswatini \| \| Lesotho \| \| Namibia \| \| South Africa \| \| Benin \| \| Burkina Faso \| \| Cabo Verde \| \| Côte d’Ivoire \| \| Gambia \| \| Ghana \| \| Guinea \| \| Guinea-Bissau \| \| Liberia \| \| Mali \| \| Mauritania \| \| Niger \| \| Nigeria \| \| Saint Helena \| \| Senegal \| \| Sierra Leone \| \| Togo \| |
| Latin America and the Caribbean | \| Anguilla \| \| --- \| \| Antigua and Barbuda \| \| Aruba \| \| Bahamas \| \| Barbados \| \| Bonaire, Sint Eustatius and Saba \| \| British Virgin Islands \| \| Cayman Islands \| \| Cuba \| \| Curaçao \| \| Dominica \| \| Dominican Republic \| \| Grenada \| \| Guadeloupe \| \| Haiti \| \| Jamaica \| \| Martinique \| \| Montserrat \| \| Puerto Rico \| \| Saint Barthélemy \| \| Saint Kitts and Nevis \| \| Saint Lucia \| \| Saint Martin (French Part) \| \| Saint Vincent and the Grenadines \| \| Sint Maarten (Dutch part) \| \| Trinidad and Tobago \| \| Turks and Caicos Islands \| \| United States Virgin Islands \| \| Belize \| \| Costa Rica \| \| El Salvador \| \| Guatemala \| \| Honduras \| \| Mexico \| \| Nicaragua \| \| Panama \| \| South America \| \| Argentina \| \| Bolivia (Plurinational State of) \| \| Bouvet Island \| \| Brazil \| \| Chile \| \| Colombia \| \| Ecuador \| \| Falkland Islands (Malvinas) \| \| French Guiana \| \| Guyana \| \| Paraguay \| \| Peru \| \| South Georgia and the South Sandwich Islands \| \| Suriname \| \| Uruguay \| \| Venezuela (Bolivarian Republic of) \| |
| Northern America | \| Bermuda \| \| --- \| \| Canada \| \| Greenland \| \| Saint Pierre and Miquelon \| \| United States of America \| \| Antarctica \| |
| Eastern-Central Asia | \| Kazakhstan \| \| --- \| \| Kyrgyzstan \| \| Tajikistan \| \| Turkmenistan \| \| Uzbekistan \| \| China \| \| China, Hong Kong Special Administrative Region \| \| China, Macao Special Administrative Region \| \| Democratic People's Republic of Korea \| \| Japan \| \| Mongolia \| \| Republic of Korea \| |
| South-eastern Asia | \| Brunei Darussalam \| \| --- \| \| Cambodia \| \| Indonesia \| \| Lao People's Democratic Republic \| \| Malaysia \| \| Myanmar \| \| Philippines \| \| Singapore \| \| Thailand \| \| Timor-Leste \| \| Viet Nam \| |
| Southern Asia | \| Afghanistan \| \| --- \| \| Bangladesh \| \| Bhutan \| \| India \| \| Iran (Islamic Republic of) \| \| Maldives \| \| Nepal \| \| Pakistan \| \| Sri Lanka \| |
| Western Asia (excluding Qatar) | \| Armenia \| \| --- \| \| Azerbaijan \| \| Bahrain \| \| Cyprus \| \| Georgia \| \| Iraq \| \| Israel \| \| Jordan \| \| Kuwait \| \| Lebanon \| \| Oman \| \| Saudi Arabia \| \| State of Palestine \| \| Syrian Arab Republic \| \| Turkey \| \| United Arab Emirates \| \| Yemen \| |
| Eastern Europe | \| Belarus \| \| --- \| \| Bulgaria \| \| Czechia \| \| Hungary \| \| Poland \| \| Republic of Moldova \| \| Romania \| \| Russian Federation \| \| Slovakia \| \| Ukraine \| |
| Northern Europe | \| Åland Islands \| \| --- \| \| Channel Islands \| \| Guernsey \| \| Jersey \| \| Sark \| \| Denmark \| \| Estonia \| \| Faroe Islands \| \| Finland \| \| Iceland \| \| Ireland \| \| Isle of Man \| \| Latvia \| \| Lithuania \| \| Norway \| \| Svalbard and Jan Mayen Islands \| \| Sweden \| \| United Kingdom of Great Britain and Northern Ireland \| |
| Southern Europe | \| Albania \| \| --- \| \| Andorra \| \| Bosnia and Herzegovina \| \| Croatia \| \| Gibraltar \| \| Greece \| \| Holy See \| \| Italy \| \| Malta \| \| Montenegro \| \| Portugal \| \| San Marino \| \| Serbia \| \| Slovenia \| \| Spain \| \| The former Yugoslav Republic of Macedonia \| |
| Western Europe | \| Austria \| \| --- \| \| Belgium \| \| France \| \| Germany \| \| Liechtenstein \| \| Luxembourg \| \| Monaco \| \| Netherlands \| \| Switzerland \| |
| Australasia | \| Australia \| \| --- \| \| Christmas Island \| \| Cocos (Keeling) Islands \| \| Heard Island and McDonald Islands \| \| New Zealand \| \| Norfolk Island \| \| Fiji \| \| New Caledonia \| \| Papua New Guinea \| \| Solomon Islands \| \| Vanuatu \| \| Guam \| \| Kiribati \| \| Marshall Islands \| \| Micronesia (Federated States of) \| \| Nauru \| \| Northern Mariana Islands \| \| Palau \| \| United States Minor Outlying Islands \| \| American Samoa \| \| Cook Islands \| \| French Polynesia \| \| Niue \| \| Pitcairn \| \| Samoa \| \| Tokelau \| \| Tonga \| \| Tuvalu \| \| Wallis and Futuna Islands \| |

Table B: Medications prescribed by diagnosis and age

| **Diagnosis** | **Medication** | **18-29** | **18-29** | **30-39** | **30-39** | **40-49** | **40-49** | **50-59** | **50-59** | **60+** | **60+** |
| --- | --- | --- | --- | --- | --- | --- | --- | --- | --- | --- | --- |
|  |  | **(N)** | **(%)** | **(N)** | **(%)** | **(N)** | **(%)** | **(N)** | **(%)** | **(N)** | **(%)** |
| Hypertension | Methyldopa | 45 | 0.7 | 348 | 0.7 | 224 | 0.1 | 25 | 0.0 | 56 | 0.0 |
|  | Clonidine and analogues | 5 | 0.1 | 30 | 0.1 | 80 | 0.0 | 251 | 0.1 | 483 | 0.2 |
|  | Thiazides, plain | 13 | 0.2 | 72 | 0.1 | 355 | 0.2 | 647 | 0.2 | 951 | 0.3 |
|  | Beta blocking agents, plain, non-selective | 173 | 2.7 | 371 | 0.7 | 413 | 0.2 | 377 | 0.1 | 320 | 0.1 |
|  | Beta blocking agents, plain, selective | 101 | 1.6 | 1204 | 2.3 | 4511 | 2.6 | 10151 | 3.4 | 15369 | 5.0 |
|  | Alpha and beta blocking agents | 23 | 0.4 | 91 | 0.2 | 264 | 0.2 | 807 | 0.3 | 1993 | 0.6 |
|  | Beta blocking agents, selective, and thiazides | 7 | 0.1 | 32 | 0.1 | 177 | 0.1 | 851 | 0.3 | 1241 | 0.4 |
|  | Dihydropyridine derivatives | 220 | 3.4 | 3196 | 6.1 | 10757 | 6.2 | 18335 | 6.2 | 22848 | 7.4 |
|  | Angiotensin-converting enzyme inhibitors, plain | 329 | 5.1 | 4509 | 8.5 | 16452 | 9.5 | 25099 | 8.5 | 23020 | 7.4 |
|  | Angiotensin-converting enzyme inhibitors and diuretics | 46 | 0.7 | 1063 | 2.0 | 4554 | 2.6 | 9120 | 3.1 | 9680 | 3.1 |
|  | Angiotensin-converting enzyme inhibitors and calcium channel blockers | 68 | 1.1 | 1126 | 2.1 | 3991 | 2.3 | 5826 | 2.0 | 4876 | 1.6 |
|  | Angiotensin II antagonists, plain | 147 | 2.3 | 2234 | 4.2 | 8151 | 4.7 | 14631 | 4.9 | 15546 | 5.0 |
|  | Angiotensin II antagonists and diuretics | 18 | 0.3 | 879 | 1.7 | 3825 | 2.2 | 7125 | 2.4 | 9722 | 3.1 |
|  | Angiotensin II antagonists and calcium channel blockers | 58 | 0.9 | 845 | 1.6 | 3179 | 1.8 | 4884 | 1.6 | 5744 | 1.9 |
|  | Sulfonamides, plain (high ceiling diuretics) | 28 | 0.4 | 435 | 0.8 | 2250 | 1.3 | 5183 | 1.8 | 7247 | 2.3 |
|  | Sulfonamides, plain (low ceiling diuretics) | 10 | 0.2 | 70 | 0.1 | 220 | 0.1 | 629 | 0.2 | 2850 | 0.9 |
|  | **Total** | **1291** | **20.0** | **16505** | **31.3** | **59403** | **34.5** | **103941** | **35.1** | **121946** | **39.4** |
| T2DM | Insulins and analogues for injection, fast-acting | 764 | 11.8 | 1297 | 2.5 | 1702 | 1.0 | 3501 | 1.2 | 4330 | 1.4 |
|  | Insulins and analogues for injection, intermediate-acting | 85 | 1.3 | 282 | 0.5 | 415 | 0.2 | 826 | 0.3 | 1080 | 0.3 |
|  | Insulins and analogues for injection, intermediate-acting combined with fast-acting | 211 | 3.3 | 742 | 1.4 | 1854 | 1.1 | 4312 | 1.5 | 6567 | 2.1 |
|  | Insulins and analogues for injection, long-acting | 805 | 12.4 | 2149 | 4.1 | 4770 | 2.8 | 9643 | 3.3 | 11090 | 3.6 |
|  | Biguanides | 1919 | 29.7 | 10665 | 20.2 | 26715 | 15.5 | 39919 | 13.5 | 36472 | 11.8 |
|  | Sulfonamides, urea derivatives | 345 | 5.3 | 5688 | 10.8 | 19961 | 11.6 | 32486 | 11.0 | 30455 | 9.8 |
|  | Combinations of oral blood glucose lowering drugs | 410 | 6.3 | 4993 | 9.5 | 15387 | 8.9 | 22931 | 7.7 | 18619 | 6.0 |
|  | Alpha glucosidase inhibitors | 0 | 0.0 | 4 | 0.0 | 35 | 0.0 | 103 | 0.0 | 166 | 0.1 |
|  | Thiazolidinediones | 28 | 0.4 | 484 | 0.9 | 2340 | 1.4 | 4585 | 1.5 | 3968 | 1.3 |
|  | Dipeptidyl peptidase 4 (dpp-4) inhibitors | 94 | 1.5 | 1324 | 2.5 | 4615 | 2.7 | 9210 | 3.1 | 10003 | 3.2 |
|  | Glucagon-like peptide-1 (GLP-1) analogues | 2 | 0.0 | 28 | 0.1 | 113 | 0.1 | 168 | 0.1 | 122 | 0.0 |
|  | Other oral blood glucose lowering drugs | 1 | 0.0 | 31 | 0.1 | 82 | 0.0 | 333 | 0.1 | 648 | 0.2 |
|  | **Total** | **4664** | **72.1** | **27687** | **52.5** | **77989** | **45.2** | **128017** | **43.2** | **123520** | **39.9** |
| Dyslipidemia | HMG-CoA reductase inhibitors | 508 | 7.9 | 8425 | 16.0 | 34464 | 20.0 | 63066 | 21.3 | 62838 | 20.3 |
|  | Fibrates | 3 | 0.0 | 25 | 0.0 | 29 | 0.0 | 56 | 0.0 | 70 | 0.0 |
|  | Other lipid modifying agents | 4 | 0.1 | 136 | 0.3 | 505 | 0.3 | 990 | 0.3 | 1044 | 0.3 |
|  | **Total** | **515** | **8** | **8586** | **16.3** | **34998** | **20.3** | **64112** | **21.6** | **63952** | **20.6** |
| **Grand total** | | **6470** |  | **52778** |  | **172390** |  | **296070** |  | **309418** |  |

Table C: Medications prescribed by gender and diagnosis

|  |  | **Female** | | **Male** | |
| --- | --- | --- | --- | --- | --- |
|  |  | **N** | **%** | **N** | **%** |
| Hypertension | Methyldopa | 661 | 0.2 | 37 | 0.0 |
|  | Clonidine and analogues | 299 | 0.1 | 550 | 0.1 |
|  | Thiazides, plain | 938 | 0.3 | 1100 | 0.2 |
|  | Beta blocking agents, plain, non-selective | 877 | 0.3 | 777 | 0.2 |
|  | Beta blocking agents, plain, selective | 14330 | 4.4 | 17006 | 3.3 |
|  | Alpha and beta blocking agents | 938 | 0.3 | 2240 | 0.4 |
|  | Beta blocking agents, selective, and thiazides | 1483 | 0.5 | 825 | 0.2 |
|  | Dihydropyridine derivatives | 22920 | 7.1 | 32436 | 6.3 |
|  | Angiotensin-converting enzyme inhibitors, plain | 22810 | 7.1 | 46599 | 9.0 |
|  | Angiotensin-converting enzyme inhibitors and diuretics | 9592 | 3.0 | 14871 | 2.9 |
|  | Angiotensin-converting enzyme inhibitors and calcium channel blockers | 5212 | 1.6 | 10675 | 2.1 |
|  | Angiotensin II antagonists, plain | 19041 | 5.9 | 21668 | 4.2 |
|  | Angiotensin II antagonists and diuretics | 10980 | 3.4 | 10589 | 2.1 |
|  | Angiotensin II antagonists and calcium channel blockers | 5424 | 1.7 | 9286 | 1.8 |
|  | Sulfonamides, plain (Low ceiling diuretics) | 7505 | 2.3 | 7638 | 1.5 |
|  | Sulfonamides, plain (High ceiling diuretics) | 1532 | 0.5 | 2247 | 0.4 |
|  | **Total** | **124542** | **38.7** | **178544** | **34.7** |
| T2DM | Insulins and analogues for injection, fast-acting | 4972 | 1.5 | 6622 | 1.3 |
|  | Insulins and analogues for injection, intermediate-acting | 1161 | 0.4 | 1527 | 0.3 |
|  | Insulins and analogues for injection, intermediate-acting combined with fast-acting | 5239 | 1.6 | 8447 | 1.6 |
|  | Insulins and analogues for injection, long-acting | 11321 | 3.5 | 17136 | 3.3 |
|  | Biguanides | 45401 | 14.1 | 70289 | 13.6 |
|  | Sulfonamides, urea derivatives | 29455 | 9.1 | 59480 | 11.5 |
|  | Combinations of oral blood glucose lowering drugs | 21557 | 6.7 | 40783 | 7.9 |
|  | Alpha glucosidase inhibitors | 118 | 0.0 | 190 | 0.0 |
|  | Thiazolidinediones | 3441 | 1.1 | 7964 | 1.5 |
|  | Dipeptidyl peptidase 4 (dpp-4) inhibitors | 8875 | 2.8 | 16371 | 3.2 |
|  | Glucagon-like peptide-1 (GLP-1) analogues | 314 | 0.1 | 119 | 0.0 |
|  | Other oral blood glucose lowering drugs | 427 | 0.1 | 668 | 0.1 |
|  | **Total** | **132281** | **41.1** | **229596** | **44.6** |
| Dyslipidemia | HMG-CoA reductase inhibitors | 64215 | 19.9 | 105086 | 20.4 |
|  | Fibrates | 83 | 0.0 | 100 | 0.0 |
|  | Other lipid modifying agents | 962 | 0.3 | 1717 | 0.3 |
|  | **Total** | **65260** | **20.2** | **106903** | **20.7** |
| **Grand total** | | **322083** |  | **515043** |  |

Table D: Medications prescribed by nationality and diagnosis

|  |  | **Qatar** | | **Northern Africa** | | **Sub-Saharan Africa** | | **Northern America** | | **South-eastern Asia** | | **Southern Asia** | | **Western Asia (excluding Qatar)** | | **All others** | |
| --- | --- | --- | --- | --- | --- | --- | --- | --- | --- | --- | --- | --- | --- | --- | --- | --- | --- |
|  |  | **N** | **%** | **N** | **%** | **N** | **%** | **N** | **%** | **N** | **%** | **N** | **%** | **N** | **%** | **N** | **%** |
| Hypertension | Methyldopa | 137 | 0.1 | 199 | 0.1 | 7 | 0.1 | 2 | 0.0 | 107 | 0.2 | 184 | 0.1 | 57 | 0.0 | 5 | 0.1 |
|  | Clonidine and analogues | 270 | 0.1 | 183 | 0.1 | 8 | 0.1 | 4 | 0.1 | 15 | 0.0 | 108 | 0.0 | 259 | 0.2 | 2 | 0.0 |
|  | Thiazides, plain | 477 | 0.3 | 386 | 0.3 | 48 | 0.6 | 13 | 0.3 | 143 | 0.3 | 527 | 0.2 | 416 | 0.3 | 28 | 0.6 |
|  | Beta blocking agents, plain, non-selective | 334 | 0.2 | 359 | 0.2 | 5 | 0.1 | 20 | 0.4 | 96 | 0.2 | 467 | 0.1 | 329 | 0.3 | 44 | 0.9 |
|  | Beta blocking agents, plain, selective | 7014 | 3.9 | 5797 | 4.0 | 303 | 3.7 | 259 | 5.4 | 1,604 | 3.7 | 10224 | 3.1 | 5,910 | 5.0 | 225 | 4.7 |
|  | Alpha and beta blocking agents | 801 | 0.4 | 599 | 0.4 | 16 | 0.2 | 60 | 1.2 | 91 | 0.2 | 894 | 0.3 | 691 | 0.6 | 26 | 0.5 |
|  | Beta blocking agents, selective, and thiazides | 487 | 0.3 | 447 | 0.3 | 24 | 0.3 | 12 | 0.2 | 169 | 0.4 | 657 | 0.2 | 512 | 0.4 | 0 | 0.0 |
|  | Dihydropyridine derivatives | 10311 | 5.7 | 9128 | 6.3 | 744 | 9.0 | 380 | 7.9 | 6,494 | 15.2 | 20151 | 6.1 | 7,754 | 6.5 | 394 | 8.3 |
|  | Angiotensin-converting enzyme inhibitors, plain | 12231 | 6.7 | 11,731 | 8.1 | 764 | 9.2 | 402 | 8.3 | 4,591 | 10.7 | 30104 | 9.1 | 9,221 | 7.7 | 365 | 7.7 |
|  | Angiotensin-converting enzyme inhibitors and diuretics | 4687 | 2.6 | 4,815 | 3.3 | 367 | 4.4 | 109 | 2.3 | 2,038 | 4.8 | 8,981 | 2.7 | 3,338 | 2.8 | 128 | 2.7 |
|  | Angiotensin-converting enzyme inhibitors and calcium channel blockers | 2115 | 1.2 | 2,882 | 2.0 | 215 | 2.6 | 108 | 2.2 | 2,002 | 4.7 | 6,556 | 2.0 | 1,916 | 1.6 | 93 | 2.0 |
|  | Angiotensin II antagonists, plain | 9400 | 5.2 | 6,126 | 4.2 | 275 | 3.3 | 276 | 5.7 | 3,237 | 7.6 | 15,095 | 4.6 | 5,970 | 5.0 | 330 | 6.9 |
|  | Angiotensin II antagonists and diuretics | 5111 | 2.8 | 3,853 | 2.6 | 205 | 2.5 | 116 | 2.4 | 1,939 | 4.5 | 6,713 | 2.0 | 3,514 | 2.9 | 118 | 2.5 |
|  | Angiotensin II antagonists and calcium channel blockers | 3088 | 1.7 | 3,025 | 2.1 | 187 | 2.3 | 89 | 1.8 | 1,466 | 3.4 | 4,420 | 1.3 | 2,279 | 1.9 | 156 | 3.3 |
|  | Sulfonamides, plain (high ceiling diuretics) | 3189 | 1.8 | 2445 | 1.7 | 169 | 2.0 | 51 | 1.1 | 1285 | 3.0 | 5600 | 1.7 | 2294 | 1.9 | 110 | 2.3 |
|  | Sulfonamides, plain (low ceiling diuretics) | 1558 | 0.9 | 493 | 0.3 | 41 | 0.5 | 20 | 0.4 | 58 | 0.1 | 708 | 0.2 | 882 | 0.7 | 19 | 0.4 |
|  | **Total** | **61210** | **33.8** | **52468** | **36.0** | **3378** | **40.8** | **1921** | **39.8** | **25335** | **59.2** | **111389** | **33.7** | **45342** | **38.0** | **2043** | **42.8** |
| T2DM | Insulins and analogues for injection, fast-acting | 3510 | 1.9 | 2622 | 1.8 | 101 | 1.2 | 99 | 2.1 | 164 | 0.4 | 3267 | 1.0 | 1788 | 1.5 | 43 | 0.9 |
|  | Insulins and analogues for injection, intermediate-acting | 821 | 0.5 | 482 | 0.3 | 43 | 0.5 | 9 | 0.2 | 43 | 0.1 | 913 | 0.3 | 374 | 0.3 | 3 | 0.1 |
|  | Insulins and analogues for injection, intermediate-acting combined with fast-acting | 4147 | 2.3 | 2403 | 1.7 | 169 | 2.0 | 25 | 0.5 | 70 | 0.2 | 5428 | 1.6 | 1386 | 1.2 | 58 | 1.2 |
|  | Insulins and analogues for injection, long-acting | 7897 | 4.4 | 5453 | 3.7 | 240 | 2.9 | 173 | 3.6 | 409 | 1.0 | 9967 | 3.0 | 4230 | 3.5 | 88 | 1.8 |
|  | Biguanides | 21340 | 11.8 | 20556 | 14.1 | 1262 | 15.2 | 524 | 10.9 | 4356 | 10.2 | 51923 | 15.7 | 15180 | 12.7 | 548 | 11.5 |
|  | Sulfonamides, urea derivatives | 18652 | 10.3 | 14623 | 10.0 | 829 | 10.0 | 297 | 6.2 | 2319 | 5.4 | 41185 | 12.5 | 10787 | 9.1 | 243 | 5.1 |
|  | Combinations of oral blood glucose lowering drugs | 15902 | 8.8 | 10995 | 7.6 | 531 | 6.4 | 317 | 6.6 | 1667 | 3.9 | 24391 | 7.4 | 8323 | 7.0 | 214 | 4.5 |
|  | Alpha glucosidase inhibitors | 121 | 0.1 | 26 | 0.0 | 0 | 0.0 | 0 | 0.0 | 5 | 0.0 | 91 | 0.0 | 65 | 0.1 | 0 | 0.0 |
|  | Thiazolidinediones | 2658 | 1.5 | 1921 | 1.3 | 99 | 1.2 | 48 | 1.0 | 140 | 0.3 | 5210 | 1.6 | 1321 | 1.1 | 8 | 0.2 |
|  | Dipeptidyl peptidase 4 (dpp-4) inhibitors | 6012 | 3.3 | 4080 | 2.8 | 266 | 3.2 | 78 | 1.6 | 431 | 1.0 | 10988 | 3.3 | 3284 | 2.8 | 107 | 2.2 |
|  | Glucagon-like peptide-1 (GLP-1) analogues | 426 | 0.2 | 1 | 0.0 | 0 | 0.0 | 0 | 0.0 | 0 | 0.0 | 0 | 0.0 | 6 | 0.0 | 0 | 0.0 |
|  | Other oral blood glucose lowering drugs | 447 | 0.2 | 195 | 0.1 | 8 | 0.1 | 7 | 0.1 | 3 | 0.0 | 258 | 0.1 | 177 | 0.1 | 0 | 0.0 |
|  | **Total** | **81933** | **45.2** | **63357** | **43.5** | **3548** | **42.8** | **1577** | **32.7** | **9607** | **22.4** | **153621** | **46.5** | **46921** | **39.4** | **1312** | **27.5** |
| Dyslipidemia | HMG-CoA reductase inhibitors | 37329 | 20.6 | 29139 | 20.0 | 1350 | 16.3 | 1279 | 26.5 | 7822 | 18.3 | 64758 | 19.6 | 26250 | 22.0 | 1374 | 28.8 |
|  | Fibrates | 46 | 0.0 | 22 | 0.0 | 1 | 0.0 | 0 | 0.0 | 2 | 0.0 | 54 | 0.0 | 57 | 0.0 | 1 | 0.0 |
|  | Other lipid modifying agents | 810 | 0.4 | 591 | 0.4 | 7 | 0.1 | 46 | 1.0 | 64 | 0.1 | 516 | 0.2 | 607 | 0.5 | 38 | 0.8 |
|  | **Total** | **38185** | **21** | **29752** | **20.4** | **1358** | **16.4** | **1325** | **27.5** | **7888** | **18.4** | **65328** | **19.8** | **26914** | **22.5** | **1413** | **29.6** |
| **Grand total** | | **181328** |  | **145577** |  | **8284** |  | **4823** |  | **42830** |  | **330338** |  | **119177** |  | **4768** |  |
